# Comparative epidemiology of five waves of COVID-19 in Mexico, March 2020–August 2022

**DOI:** 10.1186/s12879-022-07800-w

**Published:** 2022-10-31

**Authors:** Iván de Jesús Ascencio-Montiel, Oscar David Ovalle-Luna, Ramón Alberto Rascón-Pacheco, Victor Hugo Borja-Aburto, Gerardo Chowell

**Affiliations:** 1 División de Análisis en Salud. Coordinación de Vigilancia Epidemiológica, Mexican Social Security Institute, Mexico City, Mexico; 2 División de Vigilancia Epidemiológica de Enfermedades Transmisibles. Coordinación de Vigilancia Epidemiológica, Mexican Social Security Insitute, Mexico City, Mexico; 3 Coordinación de Investigación en Salud, Instituto Mexicano del Seguro Social, Mexico City, Mexico; 4 Mexican Social Security Institute, Mexico City, Mexico; 5 Department of Population Health Sciences, School of Public Health, Georgia State University, Atlanta, GA USA

**Keywords:** COVID-19, SARS-CoV-2, Hospitalization, Intubation, Death

## Abstract

**Background:**

The Mexican Institute of Social Security (IMSS) is the largest health care provider in Mexico, covering about 48% of the Mexican population. In this report, we describe the epidemiological patterns related to confirmed cases, hospitalizations, intubations, and in-hospital mortality due to COVID-19 and associated factors, during five epidemic waves recorded in the IMSS surveillance system.

**Methods:**

We analyzed COVID-19 laboratory-confirmed cases from the Online Epidemiological Surveillance System (SINOLAVE) from March 29th, 2020, to August 27th, 2022. We constructed weekly epidemic curves describing temporal patterns of confirmed cases and hospitalizations by age, gender, and wave. We also estimated hospitalization, intubation, and hospital case fatality rates. The mean days of in-hospital stay and hospital admission delay were calculated across five pandemic waves. Logistic regression models were employed to assess the association between demographic factors, comorbidities, wave, and vaccination and the risk of severe disease and in-hospital death.

**Results:**

A total of 3,396,375 laboratory-confirmed COVID-19 cases were recorded across the five waves. The introduction of rapid antigen testing at the end of 2020 increased detection and modified epidemiological estimates. Overall, 11% (95% CI 10.9, 11.1) of confirmed cases were hospitalized, 20.6% (95% CI 20.5, 20.7) of the hospitalized cases were intubated, and the hospital case fatality rate was 45.1% (95% CI 44.9, 45.3). The mean in-hospital stay was 9.11 days, and patients were admitted on average 5.07 days after symptoms onset. The most recent waves dominated by the Omicron variant had the highest incidence. Hospitalization, intubation, and mean hospitalization days decreased during subsequent waves. The in-hospital case fatality rate fluctuated across waves, reaching its highest value during the second wave in winter 2020. A notable decrease in hospitalization was observed primarily among individuals ≥ 60 years. The risk of severe disease and death was positively associated with comorbidities, age, and male gender; and declined with later waves and vaccination status.

**Conclusion:**

During the five pandemic waves, we observed an increase in the number of cases and a reduction in severity metrics. During the first three waves, the high in-hospital fatality rate was associated with hospitalization practices for critical patients with comorbidities.

**Supplementary Information:**

The online version contains supplementary material available at 10.1186/s12879-022-07800-w.

## Background

SARS-CoV-2 has spread worldwide in the form of a series of waves of infection with varying levels of transmissibility and severity [1, 2] and modulated by multiple factors, including the intrinsic transmission properties of evolving variants, social distancing measures (e.g., face mask wearing), access to medical care and vaccination campaigns. Investigating the epidemiological factors that have shaped the multi-wave COVID-19 patterns of infection, hospitalization, and death at different spatial scales could improve public health policy to save lives and minimize economic disruptions for future pandemics.

The COVID-19 pandemic has disproportionately affected many Latin American countries, with 176,521,913 cases, including 2,822,535 deaths reported as of September 8th, 2022 [3]. Mexico, one of North America’s most highly populated countries with high poverty levels [4] and low COVID-19 testing rates, documented the first imported COVID-19 case on February 27th, 2020. Mexico currently ranks 7th in the world regarding COVID-19 reported deaths, with a total of 329,652 recorded deaths and 7,046,220 reported cases (4.68% of confirmed case fatality rate) as of September 8th, 2022 [5].

In this study, we investigated the epidemiological patterns of COVID-19 infections, hospitalizations, deaths, and factors associated with severe disease outcomes during the five epidemic waves in Mexico. To that end, we used data reported by the Mexican Institute of Social Security (IMSS) surveillance system, the most extensive Latin-American social security system, and Mexico’s leading health institution [6].

## Methods

### Study design

To understand the COVID-19 epidemic in Mexico, we analyzed epidemiological surveillance data from IMSS, a tripartite Mexican health system covering active and retired workers and their families from the private sector. This group comprises roughly 60 million people, 48% of the Mexican population (126 million individuals), with a network of 1530 primary health care units and 251 2nd care level hospitals, and 25 high specialization hospitals nationwide. Overall, the IMSS population is representative of the urban Mexican population in the low-medium deciles of income. However, it is not representative of rural areas and those populations in the lowest deciles of income [6].

Epidemiological data from the Online Epidemiological Surveillance System (SINOLAVE) was used to describe trends. SINOLAVE is an epidemiological surveillance platform implemented by the IMSS during the 2009 influenza pandemic in México to collect information about acute respiratory infection cases seeking care at IMSS health care facilities [7, 8]. For the COVID-19 pandemic, SINOLAVE was adapted for COVID-19 suspected patients that fulfill the clinical signs and symptoms of the national operational case definition (during a timeframe of 10 days, presence of at least one of the following significant symptoms: fever, cough, headache, or dyspnea; accompanied by at least one of the following minor symptoms: arthralgias, myalgias odynophagia, runny nose, conjunctivitis, chest pain, anosmia, dysgeusia, and chill).

For each case, SINOLAVE collects data relating to demographic characteristics (age, gender), underlying conditions (self-reported chronic diseases such as diabetes, hypertension, COPD, cardiovascular disease, among others), symptoms at the time of testing, COVID-19 laboratory results, COVID-19 vaccination, as well as dates of symptoms onset (self-reported). Regarding vaccination status, patients were classified into four groups: (1) unvaccinated, (2) with incomplete schema (with a single dose of vaccine AstraZeneca, Sputnik, Moderna, Novavax, Pfizer BioNTech, Sinopharma, or Sinovac), (3) with complete schema (with two doses of the previous vaccines or one dose of CanSino or Janssen vaccines) and (4) with booster schema if the subject has a complete schema and an additional dose of any COVID-19 vaccine.

From the beginning and throughout the COVID-19 epidemic, the Mexican surveillance system created by the Ministry of Health established mandatory testing for 10% of symptomatic outpatient cases and 100% of hospitalized patients and deaths. Additionally, IMSS tested symptomatic active workers to issue temporal total disability certificates. However, testing coverage varied as the epidemic evolved. During the first and second waves, PCR testing was prioritized for people at high risk of severe disease (defined by age and comorbidities). Testing was available for all symptomatic cases seeking care after the introduction of rapid tests at the end of December 2020.

SINOLAVE information was supplemented with hospitalization data from the Virtual Center in Emergencies and Disasters (CVOED) platform, using an IMSS unique identification number. CVOED was used to complement case records with hospitalization and intubation status information. The latter system collects information from health care settings equipped to care for COVID-19 hospitalizations, including isolation areas and mechanical ventilation. A COVID-19 hospitalization was registered as such on SINOLAVE or CVOED. Criteria for hospitalization were based on medical judgment. Hospitalized cases frequently present with comorbidities, pneumonia, low oxygenation saturation, or respiratory failure. Additionally, a score on the scale CURB-65 (Confusion, Urea level, Respiratory rate, Blood pressure, and age ≥ 65), Pneumonia Severity Index (PSI), National Early Warning Score (NEWS) or qSOFA helped guide hospitalization decisions [9]. Since space in intensive care units was limited, hospitals were adapted to care for critical patients in general hospitalization wards in designated COVID-19 hospitals. These areas were equipped with oxygen supply, mechanical ventilation, and a health care team for every 24 patients. The team was led by a medical specialist in internal medicine, pneumology, infectiology, intensive medicine, or anesthesiology with help from other specialists or general practitioners’ doctors and by a group of nurses. These designated hospitals grew their capacity as the demand increased and have maintained a varying number of beds over time.

We included laboratory-confirmed COVID-19 cases by RT-PCR or Rapid Antigen tests (RAT) with the onset of symptoms from March 29th, 2020, to August 27th, 2022. Testing coverage varied as the epidemic evolved. During the first and second waves, PCR testing was prioritized for people at high risk of severe disease (defined by age and comorbidities). Testing was available for all suspected cases after the introduction of rapid tests at the end of 2020.

*Days of admission delay* were defined as the number of days elapsed from symptoms onset to hospitalization. *In-hospital* *days* were defined as the number of days from hospital admission to discharge or death.

Additionally, to describe the temporal distribution of SARS-CoV-2 variants, we analyzed a subset of 18,839 genetically sequenced samples. This subset with variant information included cases with onset of symptoms from April 4th, 2021, to August 20, 2022.

### Statistical analysis

#### Epidemic curves construction

Epidemic curves of the weekly rates of total confirmed and hospitalized cases were constructed from March 29th, 2020 (week 2020-14) to August 27th, 2022 (week 2022-34). We also displayed the weekly hospitalization percentage among confirmed cases, mechanical ventilation (intubated) cases among those hospitalized, and the in-hospital case fatality rate. Weekly periods were defined based on the dates of onset of symptoms. Epidemic waves correspond to the following onset of symptoms periods: the *first wave* from week 2020-14 to week 2020-40 (from March 29th, 2020 to October 3rd, 2020); the *second wave* from week 2020-41 until week 2021-21 (from October 4th, 2020 to May 29th, 2021); the *third wave* from week 2021-22 to week 2021-50 (from May 30th, 2021 until December 18th, 2021); the *fourth wave* from week 2021-51 to 2022-17 (from December 19th, 2021 to April 30th, 2022); and *fifth wave* from week 2022-18 to week 2022-34 (from May 1st, 2022 until August 27th, 2022).

To estimate confirmed incidence rates, we used the IMSS-affiliated population in June 2022 (n = 60,293,938) as the denominator (Additional file 1: Table S1).

#### Epidemic wave summary statistics

We analyzed the epidemiological characteristics across five epidemic waves occurring during summer and winter 2020, summer and winter 2021, and summer 2022, respectively (Additional file 1: Table S2). Hospitalization percentage among confirmed cases, intubation percentages among hospitalized, hospital case fatality rate, mean days of hospital admission delay, and mean days of in-hospital stay were calculated in each epidemic wave with a 95% confidence interval (95% CI). Stratified analyses by gender and age group (< 20, 20 to 39, 40 to 50, and 60 or more years old) were also carried out.

#### Factors associated with COVID-19 severe outcomes

To investigate the influence of hospitalization practices, healthcare resources-related factors, the vulnerability of the patients, and vaccination coverage on the in-hospital case fatality rate observed during the pandemic, we evaluated temporal patterns of PCR and RAT tests, the proportion of hospitalizations with comorbidities, and vaccination coverage. Multiple logistic regression models were used to assess the association of age, sex, comorbidities, vaccination status, mechanical ventilation, ICU admission, and indicator variables for the number of waves with the risk of hospitalization among all positive cases and the risk of death among hospitalized patients.

Statistical analyses were carried out using Stata version 14. Confidence intervals were calculated for exact proportions and means, assuming a normal distribution. To compare the distribution of variables among comparison groups, we used a t-test, one-way ANOVA, and Chi-square trend test.

## Results

IMSS confirmed the first case at the end of March 2020. The epidemiological system reported symptomatic and laboratory-confirmed cases. The number of cases and estimates of hospitalization and in-hospital case fatality rates are presented in Table 1.

**Table 1:** Summary of COVID-19 cases and estimates by epidemic wave

Variable	1st wave (Summer 2020)	2nd wave (Winter 2020)	3rd wave (Summer 2021)	4th wave (Winter 2021)	5th wave (Summer 2022)	All waves
Number of cases						
Suspected cases (A)	839,120	1,789,867	2,220,692	2,271,708	1,662,178	8,783,565
Suspected cases with laboratory test (B)	475,238	1,364,011	2,118,935	1,988,949	1,555,062	7,502,195
Confirmed cases (C)	264,792	549,962	762,884	1,047,624	771,113	3,396,375
Hospitalized cases (D)	105,681	143,977	75,836	34,648	13,504	373,646
Intubated cases (E)	27,437	29,899	15,078	3,804	711	76,929
In-hospital deaths (F)	47,835	73,160	33,088	12,021	2395	168,499
Estimations						
Testing percentage among suspected cases [B/Ax100] % (95% CI)*	**56.6 (56.5, 56.7)**	**76.2 (76.1, 76.3)**	**95.4 (95.4, 95.4)**	**87.6 (87.5, 87.6)**	**93.6 (93.5, 93.6)**	85.4 (85.4, 85.4)
Positivity percentage among tested cases [C/Bx100] % (95% CI)*	**55.7 (55.6, 55.9)**	**40.3 (40.2, 40.4)**	**36 (35.9, 36.1)**	**52.7 (52.6, 52.7)**	**49.6 (49.5, 49.7)**	45.3 (45.2, 45.3)
Hospitalization among confirmed cases [D/Cx100] % (95% CI)*	**39.9 (39.7, 40.1)**	**26.2 (26.1, 26.3)**	**9.9 (9.9, 10)**	**3.3 (3.3, 3.3)**	**1.8 (1.7, 1.8)**	11.0 (10.9, 11.1)
Intubation among hospitalized [E/Dx100] % (95% CI)*	**26.0 (25.7, 26.2)**	**20.8 (20.6, 21)**	**19.9 (19.6, 20.2)**	**11 (10.7, 11.3)**	**5.3 (4.9, 5.7)**	20.6 (20.5, 20.7)
Hospital case fatality rate [F/Dx100] % (95% CI)*	**45.3 (45.0, 45.6)**	**50.8 (50.6, 51.1)**	**43.6 (43.3, 44)**	**34.7 (34.2, 35.2)**	**17.7 (17.1, 18.4)**	45.1 (44.9, 45.3)
Mean days of hospital admission delay (95% CI)**	**4.55 (4.52, 4.57)**	**5.75 (5.73, 5.77)**	**5.79 (5.76, 5.82)**	**3.48 (3.44, 3.52)**	**2.02 (1.98, 2.06)**	5.07 (5.06,5.09)
Mean in-hospital days (95% CI)**	**9.59 (9.54, 9.63)**	**9.33 (9.29, 9.37)**	**9.09 (9.04, 9.15)**	**7.83 (7.75, 7.9)**	**6.41 (6.31, 6.51)**	9.11 (9.09, 9.13)

Epidemic waves correspond to the following onset of symptoms periods: the *first* wave from week 2020-14 to week 2020-40 (from March 29th, 2020 to October 3rd, 2020); the *second* wave from week 2020-41 until week 2021-21 (from October 4th, 2020 to May 29th, 2021); the *third* wave from week 2021-22 to week 2021-50 (from May 30th, 2021 until December 18th, 2021); the *fourth* wave from week 2021-51 to 2022-17 (from December 19th, 2021 to April 30th, 2022); and *fifth* wave from week 2022-18 to week 2022-34 (from May 1st, 2022 until August 27th, 2022)

Estimations with 95% confidence intervals (95%CI) are shown. *p < 0.001 by Chi-square trend test. **p < 0.001 by one-way ANOVA. Statistically significant results are shown in bold text

The five waves comprised 8,783,565 suspected cases, of which 7,502,195 were tested (85.4%) and 3,396,375 laboratory-confirmed COVID-19 cases with onset of symptoms from March 29th, 2020, to August 27th, 2022 were analyzed (positivity rate of 45.3% among tested cases). Overall, 11% cases were hospitalized (373,646 of 3,396,375 confirmed cases), 20.6% were intubated (76,929 of 373,646 hospitalized cases), and 45.1% was the overall hospital case fatality rate (168,499 of 373,646 hospitalized cases). Below we describe the epidemiological patterns related to case incidence rates of confirmed cases, hospitalizations, in-hospital mortality, hospital admission delays, and in-hospital stays across waves, age, and gender.

### Lab-confirmed COVID-19 cases

**Fig. 1:**
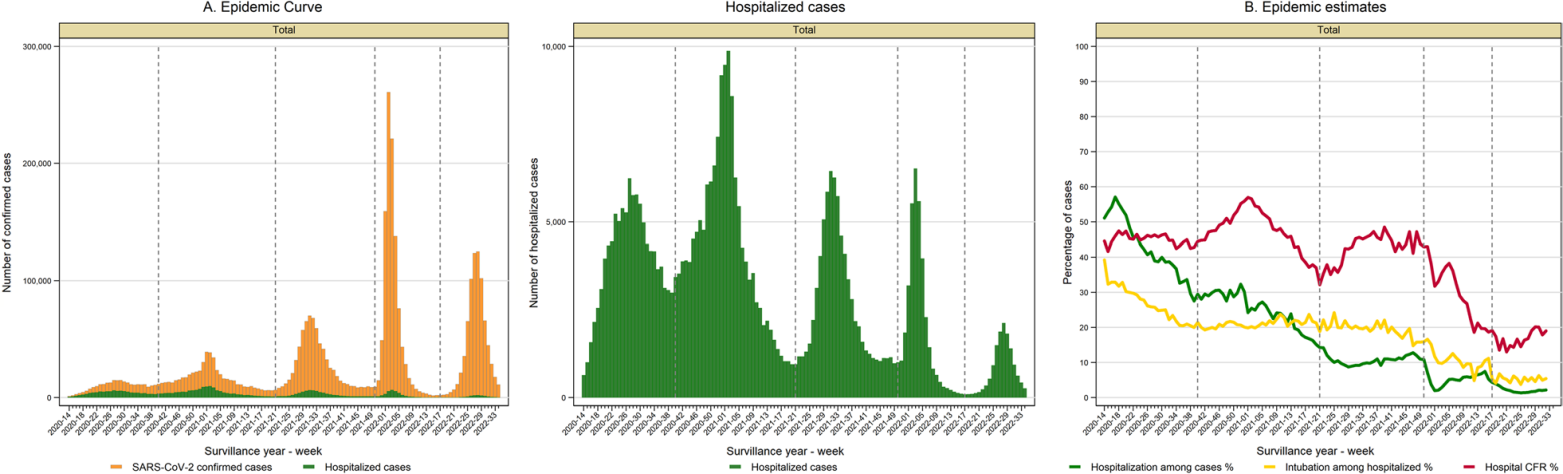
COVID-19 epidemic curve and estimates. **A** Shows the number of COVID-19 confirmed cases (orange bars for total cases and dark green bars for hospitalized cases). The middle panel shows the number of hospitalization cases in dark green bars. **B** Contains the weekly trend of the following estimations: hospitalization percentage among confirmed cases (green line), intubation percentage among hospitalized (yellow line) and hospital case fatality rate (CFR) (red line). Figures include the onset of symptoms week period from 2020-14 (April 1st, 2020) to 2022-34 (August 27th, 2022). Dotted vertical lines represent the separation of the five epidemic waves. Epidemic waves correspond to the following onset of symptoms periods: the *first* wave from week 2020-14 to week 2020-40 (from March 29th, 2020 to October 3rd, 2020); the *second* wave from week 2020-41 until week 2021-21 (from October 4th, 2020 to May 29th, 2021); the *third* wave from week 2021-22 to week 2021-50 (from May 30th, 2021 until December 18th, 2021); the *fourth* wave from week 2021-51 to 2022-17 (from December 19th, 2021 to April 30th, 2022); and *fifth* wave from week 2022-18 to week 2022-34 (from May 1st, 2022 until August 27th, 2022)

**Fig. 2:**
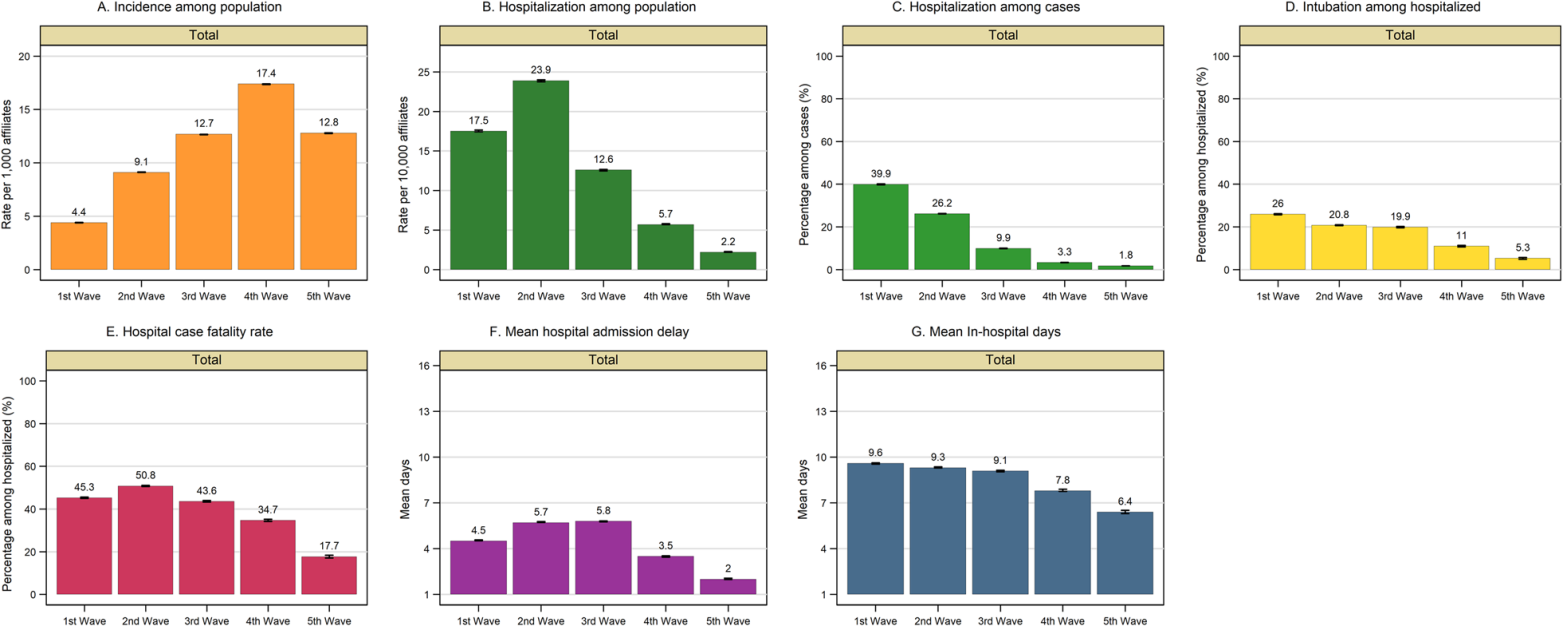
COVID-19 estimates according to the five epidemic waves. The figure shows the following estimations with a 95% confidence interval: incidence rate among the population (orange bars), hospitalization rate among the population (dark green bars), hospitalization percentage among confirmed cases (green bars), hospital case fatality rate (red bars), mean hospital admission delay (purple bars) intubation percentage (yellow bars), and mean hospitalization days (blue bars). Epidemic waves correspond to the following onset of symptoms periods: the *first* wave from week 2020-14 to week 2020-40 (from March 29th, 2020 to October 3rd, 2020); the *second* wave from week 2020-41 until week 2021-21 (from October 4th, 2020 to May 29th, 2021); the *third* wave from week 2021-22 to week 2021-50 (from May 30th, 2021 until December 18th, 2021); the *fourth* wave from week 2021-51 to 2022-17 (from December 19th, 2021 to April 30th, 2022); and *fifth* wave from week 2022-18 to week 2022-34 (from May 1st, 2022 until August 27th, 2022)

The number of weekly cases showed five peaks during the study period. The peak that occurred during January 2022 had the highest incidence, followed by the fifth peak in summer 2022. The first peak in 2020 had the lowest incidence (Figs. 1A and 2A).

Based on a subset of cases with variant information available since spring 2021, we found that the second, third, fourth, and fifth waves were dominated by the Alpha-Gamma, Delta, and Omicron variants, respectively (Additional file 1: Figure S1).

The multi-wave pattern was also evident when the epidemic curves were stratified by gender, and age groups, especially the curves for those younger than 60 years. Also, the incidence rate among males was higher during the first three waves than among females. In contrast, during the fourth and fifth waves, the incidence among females was higher than that for males. Across age groups, we found that individuals 20–39 and 40–59 years old showed the highest incidence rates during the last two waves and the first two epidemic waves, respectively. Individuals ≥ 60 years exhibited their highest incidence during the second wave in the winter of 2020 (Additional file 1: Tables S3 and S4, and Figures S2A, S3A, S4A, and S5A).

### Hospitalization rates among the population

Regarding hospitalization, the second wave showed the highest rate, followed by the first wave. Gradually, the hospitalization rates declined from the third to fifth waves (Figs. 1 and 2). Compared to females, males showed higher hospitalization rates than females. Individuals ≥ 60 years exhibited higher hospitalization rates than younger groups having their highest value during the second wave in the winter of 2020 (Additional file 1: Figures S2A, S3B, S4A, and S5B).

### Hospitalization among confirmed cases

**Fig. 3:**
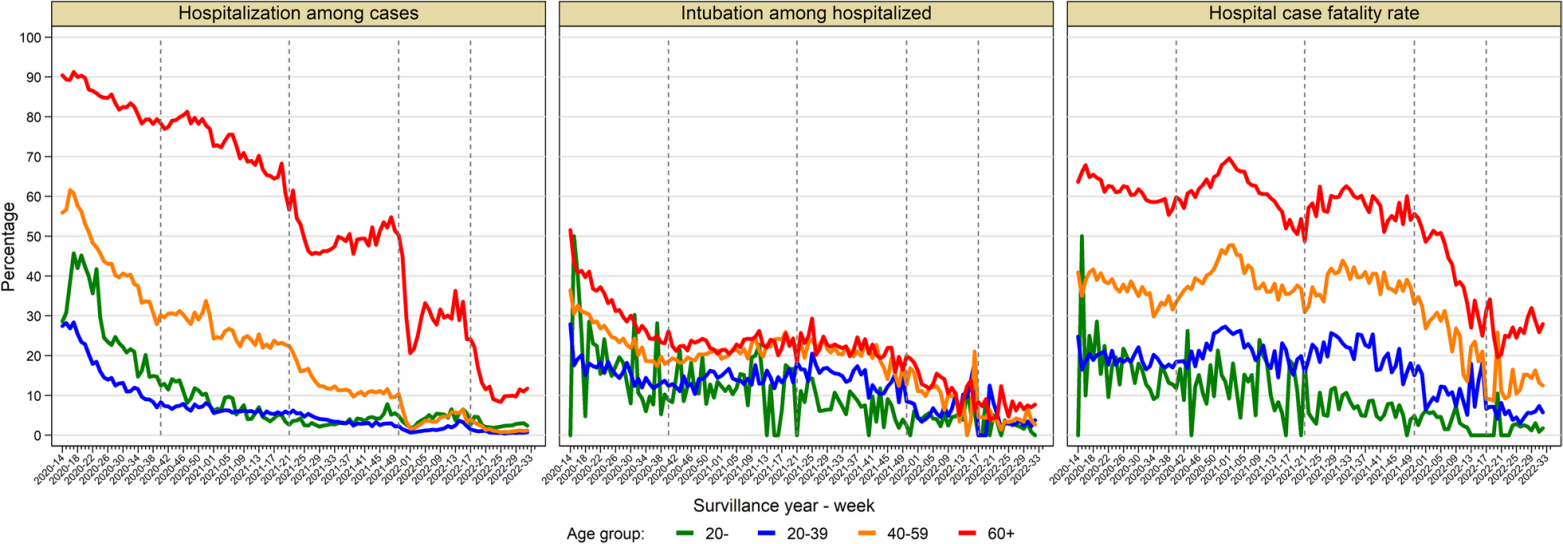
Severe COVID-19 outcomes according to age group. The figure shows the weekly trend of the following severe COVID-19 outcomes by age group: hospitalization percentage among confirmed cases (left panel), intubation percentage among hospitalized (middle panel) and hospital case fatality rate (right panel). Age groups are represented as follows: age below 20 years old in the green line, 20 to 39 years old in the blue line, 40 to 59 years in the orange line, and red line for the group aged 60 years old and over. Figures include the onset of symptoms from week 2020-14 to week 2022-34 (from April 1st, 2020, to August 27th, 2022). Dotted vertical lines represent the separation of the five epidemic waves. Epidemic waves correspond to the following onset of symptoms periods: the *first* wave from week 2020-14 to week 2020-40 (from March 29th, 2020 to October 3rd, 2020); the *second* wave from week 2020-41 until week 2021-21 (from October 4th, 2020 to May 29th, 2021); the *third* wave from week 2021-22 to week 2021-50 (from May 30th, 2021 until December 18th, 2021); the *fourth* wave from week 2021-51 to 2022-17 (from December 19th, 2021 to April 30th, 2022); and *fifth* wave from week 2022-18 to week 2022-34 (from May 1st, 2022 until August 27th, 2022)

Overall, 11% (95% CI 10.9%, 11.1%) of the cases were hospitalized. The weekly hospitalization percentage declined across waves from 39.9% (95% CI 39.7%, 40.1%) in the first wave to 1.8% (95% CI 1.7%, 1.8%) in the fifth wave (Figs. 1B and 2C). Males and older individuals (≥ 60 years) were more likely to require hospitalization than females and younger groups. The hospitalization percentage by gender and age groups decreased across subsequent waves. A substantial decrease in hospitalization percentage was observed among older individuals (≥ 60 years.) during the third and fourth waves (Fig. 3, Additional file 1: Tables S3 and S4, and Figures S2B, S3C, S4B, and S5C).

### Intubation among hospitalized cases

The overall Intubation percentage was 20.6% (95% CI 20.5, 20.7) during the study period. It decreased from 26.0% (95% CI 25.7%, 26.2%) during the first wave to 5.3% (95% CI 4.9%, 5.7%) in the fifth wave (Figs. 1B and 2D). Intubation among hospitalized cases remained stable at ~ 20% during the second and third waves. Similarly, those ≥ 60 years and males were more likely to undergo intubation. The intubation percentage declined over subsequent waves across age groups and for males and females (Fig. 3, Additional file 1: Tables S3 and S4, and Figures S2B, S3D, S4B, and S5D).

### Hospital case fatality rates

The overall hospital case fatality rate was 45.1% (95% CI 44.9, 45.3) and fluctuated over time, having its highest value during the second winter 2020 epidemic wave (50.8%, 95% CI 50.6%, 51.1%) followed by a significant decrease during the third (43.6%, 95% CI 43.3%, 44.0%), fourth (34.7%, 95% CI 34.2%, 25.2%), and fifth waves (17.7%, 95% CI 17.1%, 18.4%). It correlated with confirmed cases' epidemic peaks (Figs. 1B and 2E).

The hospital case fatality rate was higher in males than females and increased with age. Individuals ≥ 60 years exhibited the highest case fatality rate (59.3%), and those aged < 20 years showed the lowest hospital case fatality rate (Fig. 3, Additional file 1: Tables S3 and S4, and Figures S2B, S3E, S4B, and S5E).

### Hospital admission delay and length of stay

The overall hospital admission delay was 5.07 (95% CI 5.06, 5.09) days, and the mean in-hospital stay was 9.11 (95% CI 9.09, 9.13) days. While the mean in-hospital stay showed a sustained decrease over time, the hospital admission delay increased from the first wave to the third wave and decreased in the last wave (Fig. 2F and G). Males had a slightly longer hospital admission delay than females (5.22 vs. 4.87 days, t-test − 24.7, P < 0.001). Similarly, the in-hospital stay tended to be longer among males than females (9.26 vs. 8.91 days, t-test − 14.8 P < 0.001). The age group 40 to 59 years old showed the highest mean of these metrics (Additional file 1: Tables S3 and S4, and Figures S3F, G, S5F, and G). Compared with survival patients, subjects that died had lower in-hospital stay (8.1 vs. 9.9 days; t-test = 75.1, P < 0.001) and slightly higher hospital admission delay (5.2 vs. 4.9 days; t-test = 27.3, P < 0.001).

### Factors associated with COVID-19 severe outcomes

During the COVID-19 epidemic, we observed a decrease in the frequency of previous medical conditions among confirmed cases. We also observed an increase in RAT during the second wave and in COVID-19 vaccination from the third to the fifth wave (Additional file 1: Figure S6).

The number of persons who reported vaccination before the onset of illness is shown in Table 2. Ambulatory confirmed cases reported previous complete vaccination in 20.9%, while 5.4% of the hospitalized cases reported being fully vaccinated. Among those hospitalized, survivors reported 5.8% being fully vaccinated, while 4.9% of non-survivors reported being fully vaccinated.

**Table 2:** COVID-19 vaccination according to the hospitalization and in-hospital death status

Hospitalization				
COVID-19 vaccination status	Hospitalized cases		No hospitalized cases	
	Number	%	Number	%
Unvaccinated	342,410	91.6	2,083,118	68.9
Any COVID-19 vaccine	31,236	8.4	939,611	31.1
Incomplete	8014	2.1	164,414	5.4
Complete	20,298	5.4	631,142	20.9
Booster	2924	0.8	144,055	4.8
Total	373,646	100.0	3,022,729	100.0

In-hospital death				
COVID-19 vaccination status	In-hospital deaths		No in-hospital deaths	
	Number	%	Number	%
Unvaccinated	156,392	92.8	186,018	90.7
Any COVID-19 vaccine	12,107	7.2	19,129	9.3
Incomplete	2982	1.8	5032	2.5
Complete	8321	4.9	11,977	5.8
Booster	804	0.5	2120	1.0
Total	168,499	100.0	205,147	100.0

For vaccination status, patients were classified as unvaccinated, with incomplete schema (with a single dose of vaccine AstraZeneca, Sputnik, Moderna, Novavax, Pfizer BioNTech, Sinopharma or Sinovac), complete schema (with two doses of the previous vaccines or one dose of CanSino or Janssen vaccines) and booster schema if the subject has a complete schema and an additional dose of any COVID-19 vaccine

Counts and percentages are shown

In a multivariate logistic regression model, the risk of hospitalization among confirmed cases was positively associated with comorbidities, age, and male gender; and inversely associated with wave progression and complete vaccination before the onset of symptoms. The risk of death during hospitalization was reduced for those patients previously vaccinated, the fourth and fifth waves, and those admitted to an intensive care unit. As expected, this risk was increased for men and patients with comorbidities and was positively associated with age and intubation (Table 3).

**Table 3:** Multivariate logistic models for severe COVID-19 outcomes

Variable	COVID-19 outcome		
	Hospitalization OR (95% CI)	Intubation OR (95% CI)	In-hospital death OR (95% CI)
Male sex	1.56 (**1.54–1.57**)	1.20 (**1.18–1.22**)	1.32 (**1.30–1.34**)
Age group			
20–	Ref.	Ref.	Ref.
20–39	0.55 (**0.54**–**0.57**)	1.41 (**1.30**–**1.54**)	2.27 (**2.07**–**2.48**)
40–59	1.86 (**1.82**–**1.90**)	2.18 (**2.01**–**2.36**)	5.12 (**4.69**–**5.59**)
60+	12.56 (**12.26**–**12.86**)	2.57 (**2.37**–**2.79**)	13.80 (**12.64**–**15.06**)
Epidemic wave			
1st wave	Ref.	Ref.	Ref.
2nd wave	0.54 (**0.53**–**0.54**)	0.74 (**0.72**–**0.75**)	1.43 (**1.40**–**1.45**)
3rd wave	0.25 (**0.25**–**0.26**)	0.76 (**0.74**–**0.78**)	1.27 (**1.24**–**1.30**)
4th wave	0.09 (**0.09**–**0.09**)	0.37 (**0.36**–**0.39**)	0.94 (**0.91**–**0.97**)
5th wave	0.04 (**0.04**–**0.04**)	0.18 (**0.16**–**0.19**)	0.40 (**0.38**–**0.42**)
Previous medical conditions			
None	Ref.	Ref.	Ref.
One	2.15 (**2.12**–**2.17**)	1.16 (**1.13**–**1.18**)	1.29 (**1.27**–**1.32**)
Two or more	3.79 (**3.74**–**3.83**)	1.15 (**1.13**–**1.18**)	1.52 (**1.49**–**1.55**)
COVID-19 vaccination			
Unvaccinated/incomplete	Ref.	Ref.	Ref.
Complete/booster	0.41 (**0.40–0.41)**	0.96 (**0.92–0.99**)	**0.75 (0.73–0.78)**
Intubation	–	–	14.37 (**14.02–14.73**)
ICU care	–	–	0.48 (**0.46–0.51**)

The results of a multivariate logistic regression model are presented. The hospitalization model included 3,396,375 confirmed cases. The intubation and in-hospital death models comprise 373,646 hospitalized patients. Adjusted Odds ratio (OR) with 95% confidence intervals (95% CI) are shown

Previous medical conditions included obesity, hypertension, diabetes, CPOD and cardiovascular disease

For vaccination status, patients were classified into four groups: (1) unvaccinated, (2) with incomplete schema (with a single dose of vaccine AstraZeneca, Sputnik, Moderna, Novavax, Pfizer BioNTech, Sinopharma or Sinovac), (3) with complete schema (with two doses of the previous vaccines or one dose of CanSino or Janssen vaccines) and (4) with booster schema if the subject has a complete schema and an additional dose of any COVID-19 vaccine

Epidemic waves correspond to the following onset of symptoms periods: the first wave from week 2020-14 to week 2020-40 (from March 29th, 2020 to October 3rd, 2020); the second wave from week 2020-41 until week 2021-21 (from October 4th, 2020 to May 29th, 2021); the third wave from week 2021-22 to week 2021-50 (from May 30th, 2021 until December 18th, 2021); the fourth wave from week 2021-51 to 2022-17 (from December 19th, 2021 to April 30th, 2022); and fifth wave from week 2022-18 to week 2022-34 (from May 1st, 2022 until August 27th, 2022)

Statistically significant results are shown in bold text

## Discussion

The COVID-19 pandemic has been characterized by a multi-wave pattern driven by multiple factors, including social control measurements, SARS-CoV-2 variants, testing practices, vaccination, and health care infrastructure. In this report, we described the epidemiological patterns of COVID-19 during five epidemic waves from surveillance data reported by IMSS, the most extensive Mexican and Latin-American social security system. Roughly, we observed an increase in reported incidence and a decrease in the severity of COVID-19 confirmed cases over time. Additionally, based on a subset of samples, we found that the second, third, fourth, and fifth waves were dominated by the Alpha-Gamma, Delta, and Omicron SARS-CoV-2 variants, respectively. During the first three waves, Mexico experienced a high in-hospital mortality rate associated with hospitalization criteria for critical patients with comorbidities. Further research is needed to improve case management during future pandemics.

Although surveillance systems do not represent all disease cases, they provide time patterns and valuable information for policy decision-making. Confirmed cases represent only a sample of all cases because not all ill persons seek medical care and have a specimen collected [10]. Additionally, the test’s sensitivity and specificity depend on the collection timing and the specimen’s quality. This under-ascertainment of COVID-19 ill persons has been evaluated with seroprevalence and excess mortality studies [11–13]. A seroprevalence survey just before vaccination began in December 2020 estimated the extent of SARS-CoV-2 infections in Mexico, regardless of disease severity and test availability, at as much as 25%, from a national representative sample [11]. The excess mortality percentage in Mexico has been estimated at 32% across the 2 pandemic years, and the number of death certificates associated with COVID-19 has been ~ 1.5 times the number of laboratory-confirmed deaths reported in the epidemiologic surveillance system [12].

In this case, trends of positive cases are representative of testing recommendations, adequately represent critically ill patients, and underrepresent ambulatory symptomatic cases. Although the recommendations for testing were similar during the five waves, the introduction of rapid testing presented a differential practice after the second wave (Additional file 1: Figure S7). Therefore, the magnitude of the first and second waves could be underestimated, and the comparison with subsequent waves may be affected by under ascertainment bias.

During the first wave in Mexico, the hospitalization percentage among 264,792 confirmed cases was 39.9% and declined to 1.8% in the fifth wave. The first figure was similar to the hospitalization percentage of 31.9% reported in March–August 2020 in the United States [14]. We also observed a higher hospitalization percentage in males and older people. In this respect, the Palmer et al. study reported that the exponential increase in COVID-19 hospitalization rates with age could be associated inversely with thymic T-cell production, which declines in males and older age [15]. In line with our results, a Denmark study observed a reduction in hospitalization rates for Omicron cases compared with Delta variant cases [16]. Our higher hospitalization rates compared with this previous study could also be explained by the Mexican testing strategy, which has been focused on severe COVID-19 cases.

Our in-hospital case fatality rate estimates were higher than other reports worldwide [17] but similar to those reported for critically ill hospitalized patients [18, 19]. A combination of factors could explain these elevated in-hospital case fatality rate estimates. These include the hospitalization practices characterized by strict admission criteria, admitting mostly critically ill cases, healthcare resources-related factors, the high vulnerability of the sick population, and the low COVID-19 vaccination coverage during the first waves (Additional file 1: Figures S6, S8, and S9). The contribution of organizational factors, the low proportion of intubated patients during hospitalization, and the reduced number of patients admitted to ICU deserve further research.

The multivariate logistic model results confirm the independent contribution of gender, age, previous medical conditions, vaccination status, and the reduction of disease severity over time. Similar to results from Milan, Italy, reported by Giacomelli et al., the hospital case fatality rate had its highest value during the second wave [20]. De la Peña reported that the case fatality rate increased with diabetes, hypertension, obesity, immunosuppression, and end-stage kidney disease. The population attributable fraction due to obesity in outpatients was 16.8% [21]. In another study, Hong et al. [22] reported that Mexico was among the countries with the highest CFRs. They also mentioned that medical resources might be important in preventing COVID-19-related deaths; however, considering the slight variation in fatality among the elderly, preventive measures such as vaccination are more critical, especially for the elderly population, to minimize the mortality rates. Olivas-Martinez et al. [23] found that the mortality rate over time was related to the availability of ICU beds, indirectly suggesting that overcrowding contributed to hospital mortality. In contrast with other studies that report an increase in COVID-19 severity with delta strain [24, 25], our study found that the third wave, mainly driven by this variant, had a lower in-hospital case fatality rate than the first two waves. These differences could be explained by the abovementioned hospitalization criteria and testing practices during the first two waves and the COVID-19 vaccination strategy that initially targeted the most vulnerable groups, leading to a decline in hospitalization rates for high-risk groups.

As expected, we found an important contribution of vaccination in preventing severe disease and reducing the risk of hospitalization. The OR for hospitalization among confirmed cases was 0.41 (95% CI 0.40, 0.41) compared to unvaccinated or incomplete vaccination. The impact of vaccination on the population level requires additional information for the general population. This information was not available in this study, restricted to laboratory-confirmed cases. However, the impact of vaccination on the COVID-19 pandemic has recently been reported with promising results [26–28].

A strength of our study is the large sample size, including records for nearly 3.4 million laboratory-confirmed COVID-19 cases during a 2-year pandemic period stemming from a population of 60 million individuals, representing the Mexican Population. Nevertheless, our findings need to be contextualized, considering some limitations. The analyzed data derives from passive database systems, which collect information from patients that seek and receive medical care at IMSS health care facilities. In this respect, milder COVID-19 cases would not be fully represented. Additionally, specific information about administering specific therapeutics before or during hospitalization was unavailable. With our data, we could not distinguish between hospitalization due to COVID-19 and hospitalizations for reasons other than COVID-19. Moreover, only vaccination coverage at the Mexican population level was available, with no information on specific coverage for IMSS affiliates.

## Conclusions

In summary, our findings indicate that reported incidence tended to increase during the five COVID-19 epidemic waves experienced by the Mexican population. Still, severity tended to decline over subsequent waves, with an important effect of vaccination. The high in-hospital mortality rate was associated with hospitalization criteria for critical patients with comorbidities during the first three waves.

## Supplementary Information

**Additional file 1.** Additional Tables S1–S4 and Figures S1–S9.

## Data Availability

The datasets used and/or analyzed during the current study are available from the corresponding author upon reasonable request.

## Abbreviations

IMSS: Mexican Institute of Social Security
IRR: Incidence rate ratio
COPD: Chronic obstructive pulmonary disease
COVID-19: Coronavirus disease 2019
CVOED: Virtual Center in Emergencies and Disasters
RR: Relative risk
RT-PCR: Real-Time Reverse transcription polymerase chain reaction
SARS-CoV-2: Severe acute respiratory syndrome coronavirus 2
SINOLAVE: Online Epidemiological Surveillance System
