# Supplementary material for: Comparative epidemiology of five waves of COVID-19 in Mexico, March 2020–August 2022
Source: BMC Infect Dis. 2022 Oct 31;22:813. doi: 10.1186/s12879-022-07800-w (PMC9623964; doi:10.1186/s12879-022-07800-w)
Supplement: Supplementary file 1 — Additional file 1. Additional Tables S1–S4 and Figures S1–S9. [file 12879_2022_7800_MOESM1_ESM.docx]

**Supplementary Information for the Article**

Comparative epidemiology of five waves of COVID-19 in Mexico, March 2020 - August 2022

**Supplementary Table 1. Affiliated population in June 2022.**

| **Age group** | **Males** | **Females** | **Males and Females** |
| --- | --- | --- | --- |
| < 20 years old | 7,205,761 | 7,013,996 | 14,219,757 |
| 20 to 39 years old | 9,073,202 | 9,669,522 | 18,742,724 |
| 40 to 59 years old | 6,880,372 | 8,620,341 | 15,500,713 |
| 60 years old or more | 5,122,880 | 6,690,930 | 11,813,810 |
| Not specified | 8,845 | 8,089 | 16,934 |
| **All age groups** | **28,291,060** | **32,002,878** | **60,293,938** |

These figures correspond to the IMSS affiliate population to Primary Care Health Unit in June 2022.

**Supplementary Table 2. Epidemic waves start and end dates and weeks.**

| **Wave** | **1st Wave** | **2nd Wave** | **3rd Wave** | **4th Wave** | **5th Wave** | **All Waves** |
| --- | --- | --- | --- | --- | --- | --- |
| **Season** | **Summer 2020** | **Winter 2020** | **Summer 2021** | **Winter 2022** | **Summer 2022** | - |
| **Dates** |  |  |  |  |  |  |
| Start | March 29th, 2020 | October 4th, 2020 | May 30th, 2021 | December 19th, 2021 | May 1st, 2022 | March 29th, 2020 |
| End | October 3rd, 2020 | May 29th, 2021 | December 18th, 2021 | April 30th, 2022 | August 27th, 2022 | August 27th, 2022 |
| Duration | 189 | 238 | 203 | 133 | 119 | 882 |
| **Weeks** |  |  |  |  |  |  |
| Start | 2020-14 | 2020-41 | 2021-22 | 2021-51 | 2022-18 | 2020-14 |
| End | 2020-40 | 2021-21 | 2021-50 | 2022-17 | 2022-34 | 2022-34 |
| Duration | 27 | 34 | 29 | 19 | 17 | 126 |

**Supplementary Table 3. Summary of COVID-19 cases and estimates by epidemic wave according to sex.**

| **Variable** | **1st Wave (Summer 2020)** | **2nd Wave (Winter 2020)** | **3rd Wave (Summer 2021)** | **4th Wave (Winter 2021)** | **5th Wave (Summer 2022)** | **All Waves** |
| --- | --- | --- | --- | --- | --- | --- |
| **Females** | | | | | | |
| **Number of cases** |  |  |  |  |  |  |
| Confirmed cases (A) | 125,510 | 269,130 | 376,933 | 586,850 | 439,875 | 1,798,298 |
| Hospitalized cases (B) | 42,105 | 60,102 | 33,616 | 16,503 | 6,935 | 159,261 |
| Intubated cases (C) | 10,033 | 11,585 | 6,350 | 1,523 | 280 | 29,771 |
| In-hospital deaths (D) | 17,658 | 28,558 | 13,735 | 4,945 | 1,004 | 65,900 |
| **Estimations** |  |  |  |  |  |  |
| Hospitalization among confirmed cases [B/Ax100] % (95% CI) | 33.5 (33.3,33.8) | 22.3 (22.2,22.5) | 8.9 (8.8,9.0) | 2.8 (2.8,2.9) | 1.6 (1.5,1.6) | 8.9 (8.8,8.9) |
| Intubation among hospitalized [C/Bx100] % (95% CI) | 23.8 (23.4,24.2) | 19.3 (19,19.6) | 18.9 (18.5,19.3) | 9.2 (8.8,9.7) | 4 (3.6,4.5) | 18.7 (18.5,18.9) |
| Hospital case fatality rate [D/Bx100] % (95% CI) | 41.9 (41.5,42.4) | 47.5 (47.1,47.9) | 40.9 (40.3,41.4) | 30 (29.3,30.7) | 14.5 (13.7,15.3) | 41.4 (41.1,41.6) |
| Mean days of hospital admission delay (95% CI) | 4.44 (4.4,4.47) | 5.57 (5.54,5.6) | 5.56 (5.52,5.61) | 3.26 (3.21,3.32) | 1.98 (1.93,2.04) | 4.87 (4.85,4.89) |
| Mean in-hospital days (95% CI) | 9.44 (9.37,9.51) | 9.19 (9.13,9.25) | 8.9 (8.82,8.98) | 7.62 (7.52,7.73) | 6.24  (6.1,6.37) | 8.91 (8.87,8.94) |
| **Males** | | | | | | |
| **Number of cases** |  |  |  |  |  |  |
| Confirmed cases (A) | 139,282 | 280,832 | 385,951 | 460,774 | 331,238 | 1,598,077 |
| Hospitalized cases (B) | 63,576 | 83,875 | 42,220 | 18,145 | 6,569 | 214,385 |
| Intubated cases (C) | 17,404 | 18,314 | 8,728 | 2,281 | 431 | 47,158 |
| In-hospital deaths (D) | 30,177 | 44,602 | 19,353 | 7,076 | 1,391 | 102,599 |
| **Estimations** |  |  |  |  |  |  |
| Hospitalization among confirmed cases [B/Ax100] % (95% CI) | 45.6 (45.4,45.9) | 29.9 (29.7,30) | 10.9 (10.8,11) | 3.9 (3.9,4.0) | 2 (1.9,2) | 13.4 (13.4,13.5) |
| Intubation among hospitalized [C/Bx100] % (95% CI) | 27.4 (27,27.7) | 21.8 (21.6,22.1) | 20.7 (20.3,21.1) | 12.6 (12.1,13.1) | 6.6 (6,7.2) | 22 (21.8,22.2) |
| Hospital case fatality rate [D/Bx100] % (95% CI) | 47.5 (47.1,47.9) | 53.2 (52.8,53.5) | 45.8 (45.4,46.3) | 39 (38.3,39.7) | 21.2 (20.2,22.2) | 47.9 (47.6,48.1) |
| Mean days of hospital admission delay (95% CI) | 4.62 (4.59,4.65) | 5.88 (5.85,5.9) | 5.97 (5.93,6.01) | 3.68 (3.63,3.74) | 2.05 (1.99,2.12) | 5.22 (5.2,5.24) |
| Mean in-hospital days (95% CI) | 9.69 (9.63,9.74) | 9.43 (9.38,9.48) | 9.25 (9.18,9.32) | 8.01 (7.91,8.11) | 6.59 (6.45,6.74) | 9.26 (9.23,9.29) |

**Supplementary Table 4. Summary of COVID-19 cases and estimates by epidemic wave according to age groups.**

| **Variable** | **1st Wave (Summer 2020)** | **2nd Wave (Winter 2020)** | **3rd Wave (Summer 2021)** | **4th Wave (Winter 2021)** | **5th Wave (Summer 2022)** | **All Waves** |
| --- | --- | --- | --- | --- | --- | --- |
| **Age below 20 years old** | | | | | | |
| **Number of cases** |  |  |  |  |  |  |
| Confirmed cases (A) | 4,566 | 71,349 | 81,087 | 73,551 | 247,741 | 71,349 |
| Hospitalized cases (B) | 1,042 | 2,268 | 2,602 | 1,853 | 9,040 | 2,268 |
| Intubated cases (C) | 167 | 168 | 118 | 46 | 657 | 168 |
| In-hospital deaths (D) | 167 | 164 | 115 | 39 | 649 | 164 |
| **Estimations** |  |  |  |  |  |  |
| Hospitalization among confirmed cases [B/Ax100] % (95% CI) | 22.8 (21.6,24.1) | 7.4 (7,7.8) | 3.2 (3.1,3.3) | 3.2 (3.1,3.3) | 2.5 (2.4,2.6) | 3.6 (3.6,3.7) |
| Intubation among hospitalized [C/Bx100] % (95% CI) | 16 (13.8,18.4) | 12.4 (10.6,14.3) | 7.4 (6.4,8.6) | 4.5 (3.8,5.4) | 2.5 (1.8,3.3) | 7.3 (6.7,7.8) |
| Hospital case fatality rate [D/Bx100] % (95% CI) | 16 (13.8,18.4) | 12.9 (11.1,14.8) | 7.2 (6.2,8.4) | 4.4 (3.7,5.3) | 2.1 (1.5,2.9) | 7.2 (6.7,7.7) |
| Mean days of hospital admission delay (95% CI) | 2.21 (2.01,2.41) | 3.2 (3,3.4) | 3.53 (3.38,3.67) | 1.99 (1.88,2.09) | 1.56 (1.47,1.65) | 2.48 (2.42,2.55) |
| Mean in-hospital days (95% CI) | 10.19 (9.63,10.75) | 8.62 (8.21,9.04) | 7.32 (7.03,7.62) | 6.63 (6.38,6.88) | 5.56  (5.3,5.83) | 7.28 (7.13,7.43) |
| **Age from 20 to 39 years old** | | | | | | |
| **Number of cases** |  |  |  |  |  |  |
| Confirmed cases (A) | 99,864 | 235,988 | 422,122 | 557,029 | 380,311 | 1,695,314 |
| Hospitalized cases (B) | 13,532 | 15,198 | 15,722 | 5,296 | 2,246 | 51,994 |
| Intubated cases (C) | 2,121 | 2,177 | 2,430 | 269 | 70 | 7,067 |
| In-hospital deaths (D) | 2,588 | 3,427 | 3,520 | 514 | 118 | 10,167 |
| **Estimations** |  |  |  |  |  |  |
| Hospitalization among confirmed cases [B/Ax100] % (95% CI) | 13.6 (13.3,13.8) | 6.4 (6.3,6.5) | 3.7 (3.7,3.8) | 1 (0.9,1) | 0.6 (0.6,0.6) | 3.1 (3,3.1) |
| Intubation among hospitalized [C/Bx100] % (95% CI) | 15.7 (15.1,16.3) | 14.3 (13.8,14.9) | 15.5 (14.9,16) | 5.1 (4.5,5.7) | 3.1 (2.4,3.9) | 13.6 (13.3,13.9) |
| Hospital case fatality rate [D/Bx100] % (95% CI) | 19.1 (18.5,19.8) | 22.5 (21.9,23.2) | 22.4 (21.7,23) | 9.7 (8.9,10.5) | 5.3 (4.4,6.3) | 19.6 (19.2,19.9) |
| Mean days of hospital admission delay (95% CI) | 4.3 (4.24,4.37) | 5.47 (5.4,5.53) | 5.87 (5.8,5.94) | 2.47 (2.38,2.56) | 1.7 (1.61,1.8) | 4.82 (4.78,4.86) |
| Mean in-hospital days (95% CI) | 9.08 (8.96,9.21) | 9  (8.89,9.12) | 8.85 (8.74,8.96) | 7.44 (7.25,7.63) | 5.78 (5.53,6.03) | 8.68 (8.62,8.75) |
| **Variable** | **1st Wave (Summer 2020)** | **2nd Wave (Winter 2020)** | **3rd Wave (Summer 2021)** | **4th Wave (Winter 2021)** | **5th Wave (Summer 2022)** | **All Waves** |
| **Age from 40 to 59 years old** | | | | | | |
| **Number of cases** |  |  |  |  |  |  |
| Confirmed cases (A) | 102,347 | 197,520 | 200,677 | 342,451 | 250,085 | 1,093,080 |
| Hospitalized cases (B) | 42,695 | 53,165 | 24,603 | 8,582 | 2,511 | 131,556 |
| Intubated cases (C) | 10,312 | 10,950 | 5,185 | 970 | 101 | 27,518 |
| In-hospital deaths (D) | 15,603 | 22,019 | 9,774 | 2,486 | 350 | 50,232 |
| **Estimations** |  |  |  |  |  |  |
| Hospitalization among confirmed cases [B/Ax100] % (95% CI) | 41.7 (41.4,42) | 26.9 (26.7,27.1) | 12.3 (12.1,12.4) | 2.5  (2.5,2.6) | 1 (1,1) | 12 (12,12.1) |
| Intubation among hospitalized [C/Bx100] % (95% CI) | 24.2 (23.7,24.6) | 20.6 (20.3,20.9) | 21.1 (20.6,21.6) | 11.3 (10.6,12) | 4 (3.3,4.9) | 20.9 (20.7,21.1) |
| Hospital case fatality rate [D/Bx100] % (95% CI) | 36.5 (36.1,37) | 41.4 (41,41.8) | 39.7 (39.1,40.3) | 29 (28,29.9) | 13.9 (12.6,15.4) | 38.2 (37.9,38.4) |
| Mean days of hospital admission delay (95% CI) | 4.61 (4.58,4.65) | 5.96 (5.92,5.99) | 6 (5.95,6.06) | 3.58 (3.5,3.66) | 1.99  (1.89,2.1) | 5.3 (5.28,5.32) |
| Mean in-hospital days (95% CI) | 9.9 (9.83,9.97) | 9.78 (9.71,9.84) | 9.53 (9.44,9.62) | 8.16 (8.01,8.3) | 6.54  (6.3,6.78) | 9.61 (9.57,9.65) |
| **Age 60 years old or more** | | | | | | |
| **Number of cases** |  |  |  |  |  |  |
| Confirmed cases (A) | 58,015 | 99,266 | 68,736 | 67,057 | 67,166 | 360,240 |
| Hospitalized cases (B) | 48,412 | 74,339 | 33,243 | 18,168 | 6,894 | 181,056 |
| Intubated cases (C) | 14,837 | 16,614 | 7,295 | 2,447 | 494 | 41,687 |
| In-hospital deaths (D) | 29,477 | 47,550 | 19,630 | 8,906 | 1,888 | 107,451 |
| **Estimations** |  |  |  |  |  |  |
| Hospitalization among confirmed cases [B/Ax100] % (95% CI) | 83.4 (83.1,83.7) | 74.9 (74.6,75.2) | 48.4 (48,48.7) | 27.1 (26.8,27.4) | 10.3 (10,10.5) | 50.3 (50.1,50.4) |
| Intubation among hospitalized [C/Bx100] % (95% CI) | 30.6 (30.2,31.1) | 22.3 (22,22.7) | 21.9 (21.5,22.4) | 13.5 (13,14) | 7.2 (6.6,7.8) | 23 (22.8,23.2) |
| Hospital case fatality rate [D/Bx100] % (95% CI) | 60.9 (60.5,61.3) | 64 (63.6,64.3) | 59.1 (58.5,59.6) | 49 (48.3,49.8) | 27.4 (26.3,28.5) | 59.3 (59.1,59.6) |
| Mean days of hospital admission delay (95% CI) | 4.6 (4.57,4.64) | 5.7 (5.67,5.73) | 5.75 (5.7,5.8) | 3.95 (3.89,4) | 2.25 (2.19,2.31) | 5.11 (5.09,5.13) |
| Mean in-hospital days (95% CI) | 9.44 (9.37,9.5) | 9.09 (9.04,9.14) | 9 (8.93,9.08) | 7.95 (7.85,8.05) | 6.8 (6.66,6.94) | 8.97 (8.93,9) |


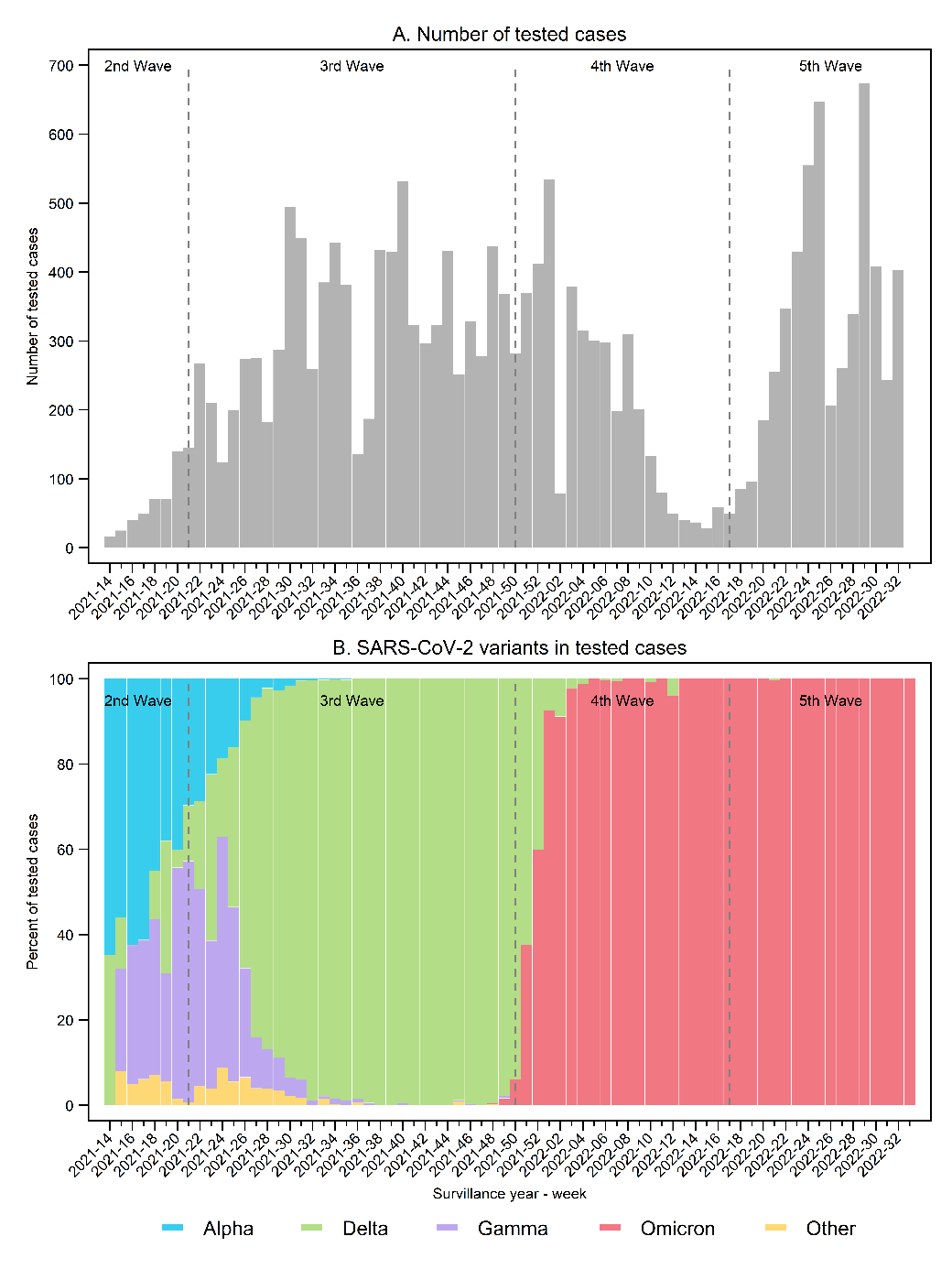


**Supplementary Figure 1. SARS-CoV-2 variant on tested cases.**

Panel A shows the weekly distribution of laboratory confirmed COVID-19 patients tested for SARS-CoV-2 variants during the second, third, fourth and fifth waves (n=18,839). Panel B contains the weekly percentage distribution of SARS-CoV-2 variants: Alpha, Gamma, Delta, Omicron and other variants (Beta, Mu, Lambda and Zeta) are shown in blue, purple, green, red and yellow bars respectively. Beta, Zeta, Mu and Lambda variants were included in the other variants category.

Figures include the onset of symptoms week period from 2021-14 (April 4th, 2021) to 2022-33 (August 20, 2022). Dotted vertical lines represent the separation of the second, third, fourth and fifth epidemic waves. In this graph, epidemic waves corresponded to the following onset of symptoms periods: the second wave from week 2021-14 until week 2021-21 (from April 4th, 2021, to May 29th, 2021); the third wave from week 2021-22 until week 2021-50 (from May 30th, 2021 until December 18th, 2021); the fourth wave from week 2021-51 to week 2022-17 (from December 19th, 2021 until April 30th, 2022); and the fitth wave from week 2022-18 to week 2022-33 (from May 1st, 2022 to August 20th, 2022).

**
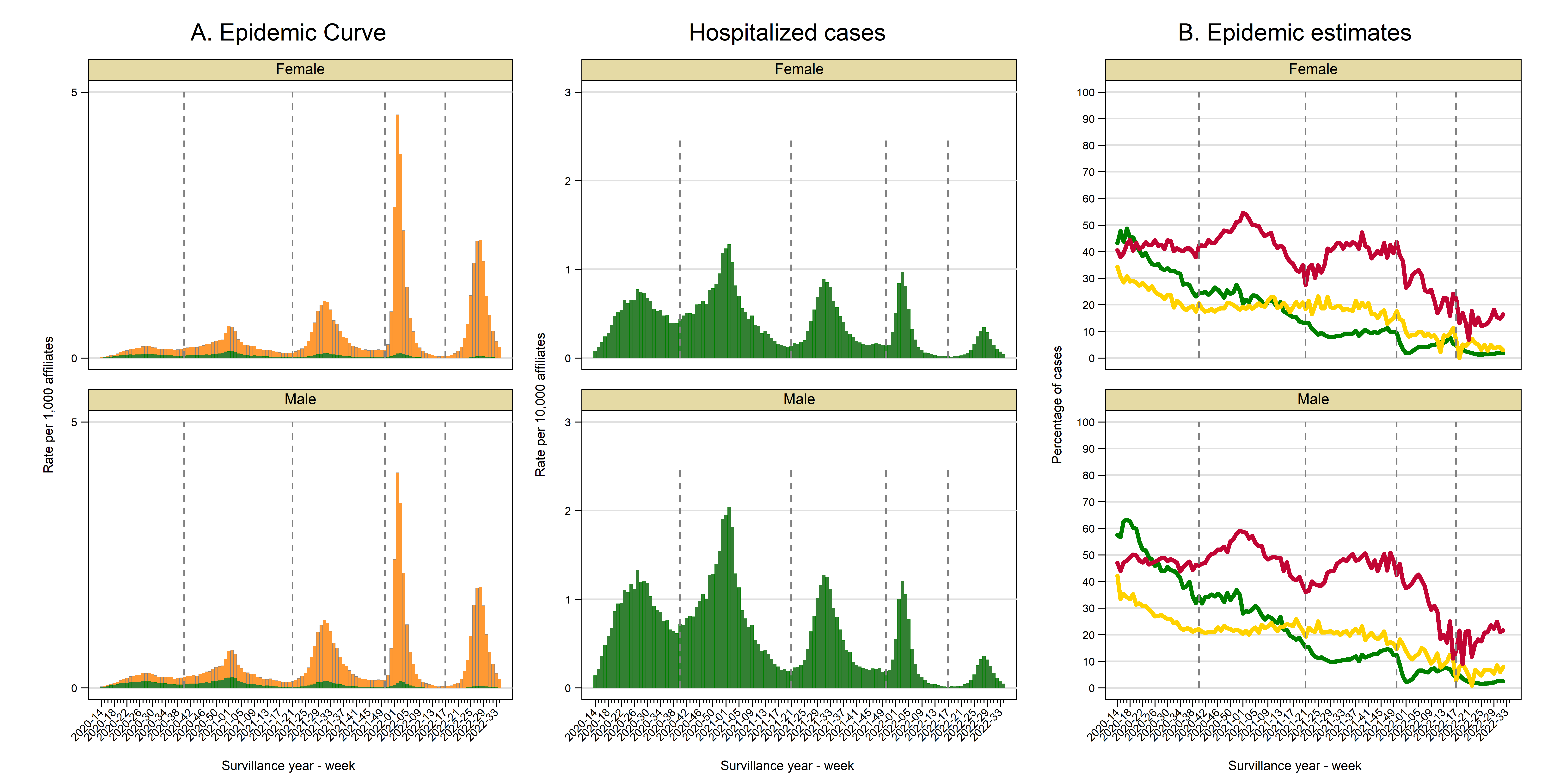
**

**Supplementary Figure 2. COVID-19 epidemic curve and estimates in males and females**

Panel A shows the COVID-19 rate of confirmed cases per 1,000 affiliates (orange bars for total cases and dark green bars for hospitalized cases). The middle panel shows the hospitalization rate among population per 10,000 affiliates in dark green bars. Panel B contains the weekly trend of the following estimations: hospitalization percentage among confirmed cases (green line), intubation percentage among hospitalized (yellow line) and hospital case fatality rate (red line).

Figures include the onset of symptoms period from week 2020-14 to week 2022-34 (from April 1st, 2020, to August 27th, 2022). Dotted vertical lines represent the separation of the five epidemic waves.

Epidemic waves correspond to the following onset of symptoms periods: the first wave from week 2020-14 to week 2020-40 (from March 29th, 2020 to October 3rd, 2020); the second wave from week 2020-41 until week 2021-21 (from October 4th, 2020 to May 29th, 2021); the third wave from week 2021-22 to week 2021-50 (from May 30th, 2021 until December 18th, 2021); the fourth wave from week 2021-51 to 2022-17 (from December 19th, 2021 to April 30th, 2022); and fifth wave from week 2022-18 to week 2022-34 (from May 1st, 2022 until August 27th, 2022).

**
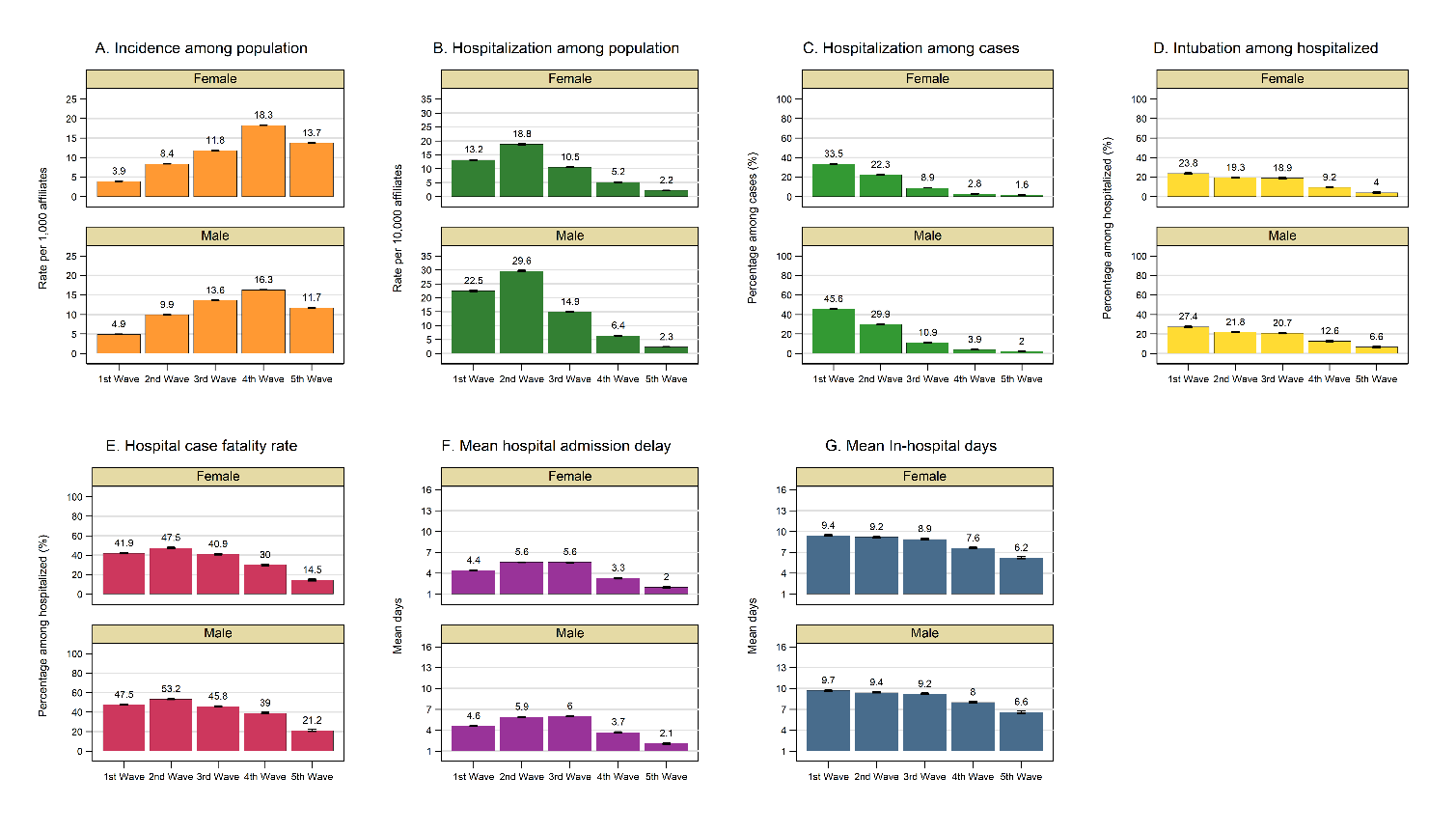
**

**Supplementary Figure 3. COVID-19 estimates according to the five epidemic waves in males and females**

The figure shows the following estimations with a 95% confidence interval: incidence rate among the population (orange bars), hospitalization rate among the population (dark green bars), hospitalization percentage among confirmed cases (green bars), intubation percentage among hospitalized (yellow bars), hospital case fatality rate (red bars), mean hospital admission delay (purple bars) and mean hospitalization days (blue bars).

Epidemic waves correspond to the following onset of symptoms periods: the first wave from week 2020-14 to week 2020-40 (from March 29th, 2020 to October 3rd, 2020); the second wave from week 2020-41 until week 2021-21 (from October 4th, 2020 to May 29th, 2021); the third wave from week 2021-22 to week 2021-50 (from May 30th, 2021 until December 18th, 2021); the fourth wave from week 2021-51 to 2022-17 (from December 19th, 2021 to April 30th, 2022); and fifth wave from week 2022-18 to week 2022-34 (from May 1st, 2022 until August 27th, 2022).


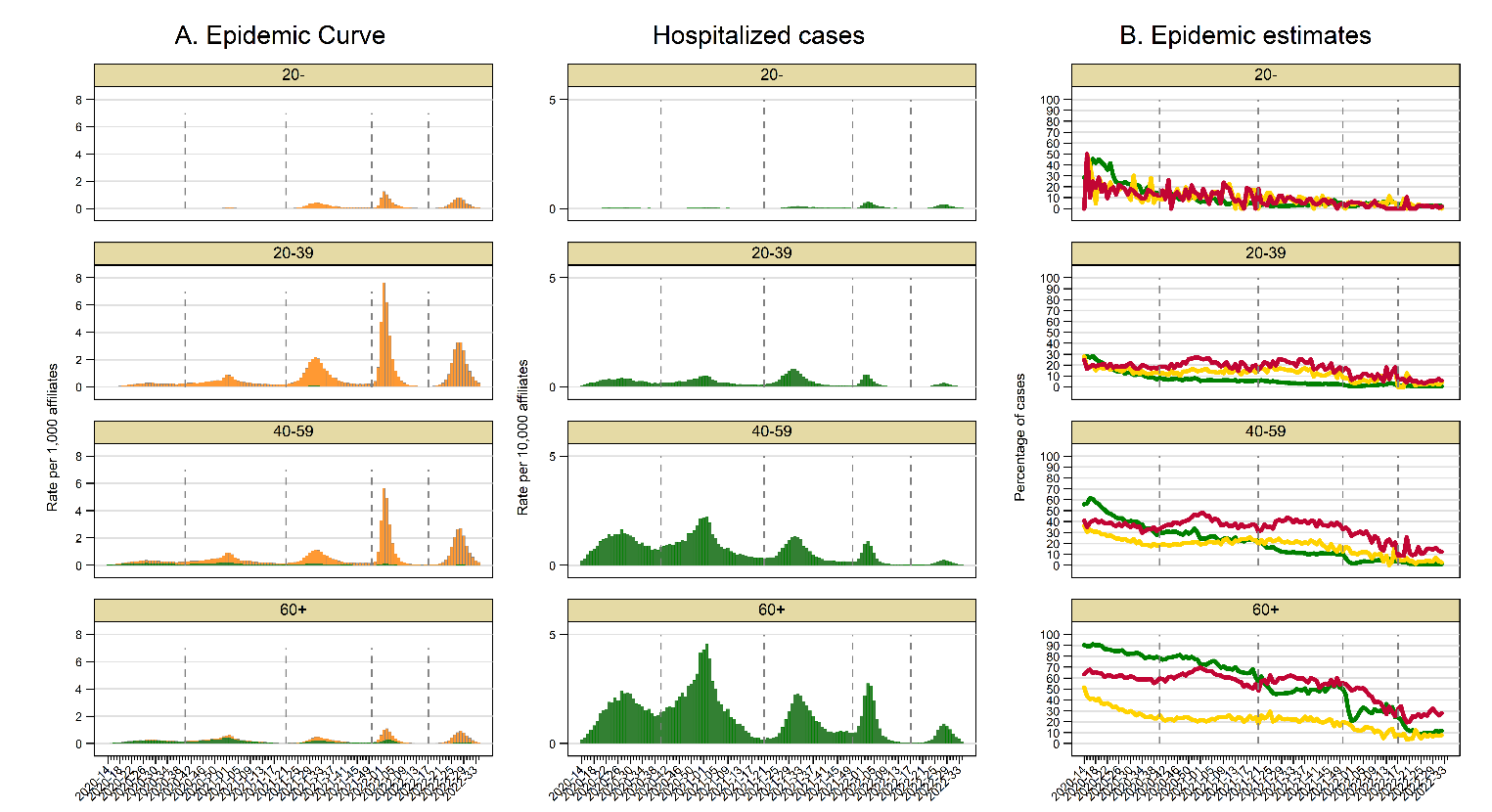


**Supplementary Figure 4. COVID-19 epidemic curve and estimates according to age group**

Panel A shows the COVID-19 rate of confirmed cases per 1,000 affiliates (orange bars for total cases and dark green bars for hospitalized cases). The middle panel shows the hospitalization rate among population per 10,000 affiliates in dark green bars. Panel B contains the weekly trend of the following estimations: hospitalization percentage among confirmed cases (green line), intubation percentage among hospitalized (yellow line) and hospital case fatality rate (red line).

Figures include the onset of symptoms period from week 2020-14 to week 2022-34 (from April 1st, 2020, to August 27th, 2022). Dotted vertical lines represent the separation of the five epidemic waves.

Epidemic waves correspond to the following onset of symptoms periods: the first wave from week 2020-14 to week 2020-40 (from March 29th, 2020 to October 3rd, 2020); the second wave from week 2020-41 until week 2021-21 (from October 4th, 2020 to May 29th, 2021); the third wave from week 2021-22 to week 2021-50 (from May 30th, 2021 until December 18th, 2021); the fourth wave from week 2021-51 to 2022-17 (from December 19th, 2021 to April 30th, 2022); and fifth wave from week 2022-18 to week 2022-34 (from May 1st, 2022 until August 27th, 2022).

**
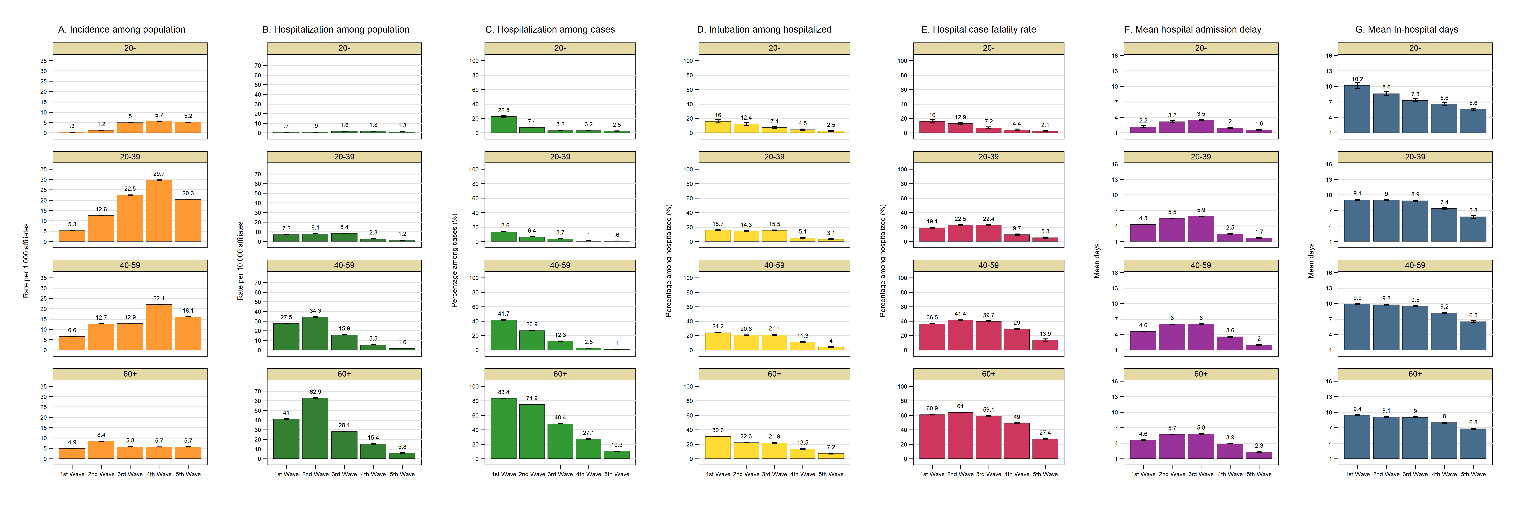
**

**Supplementary Figure 5. COVID-19 estimates across five epidemic waves by age group.**

The figure shows the following estimations with a 95% confidence interval: incidence rate among the population (orange bars), hospitalization rate among the population (dark green bars), hospitalization percentage among confirmed cases (green bars), intubation percentage among hospitalized (yellow bars), hospital case fatality rate (red bars), mean hospital admission delay (purple bars) and mean hospitalization days (blue bars).

Epidemic waves correspond to the following onset of symptoms periods: the first wave from week 2020-14 to week 2020-40 (from March 29th, 2020 to October 3rd, 2020); the second wave from week 2020-41 until week 2021-21 (from October 4th, 2020 to May 29th, 2021); the third wave from week 2021-22 to week 2021-50 (from May 30th, 2021 until December 18th, 2021); the fourth wave from week 2021-51 to 2022-17 (from December 19th, 2021 to April 30th, 2022); and fifth wave from week 2022-18 to week 2022-34 (from May 1st, 2022 until August 27th, 2022).


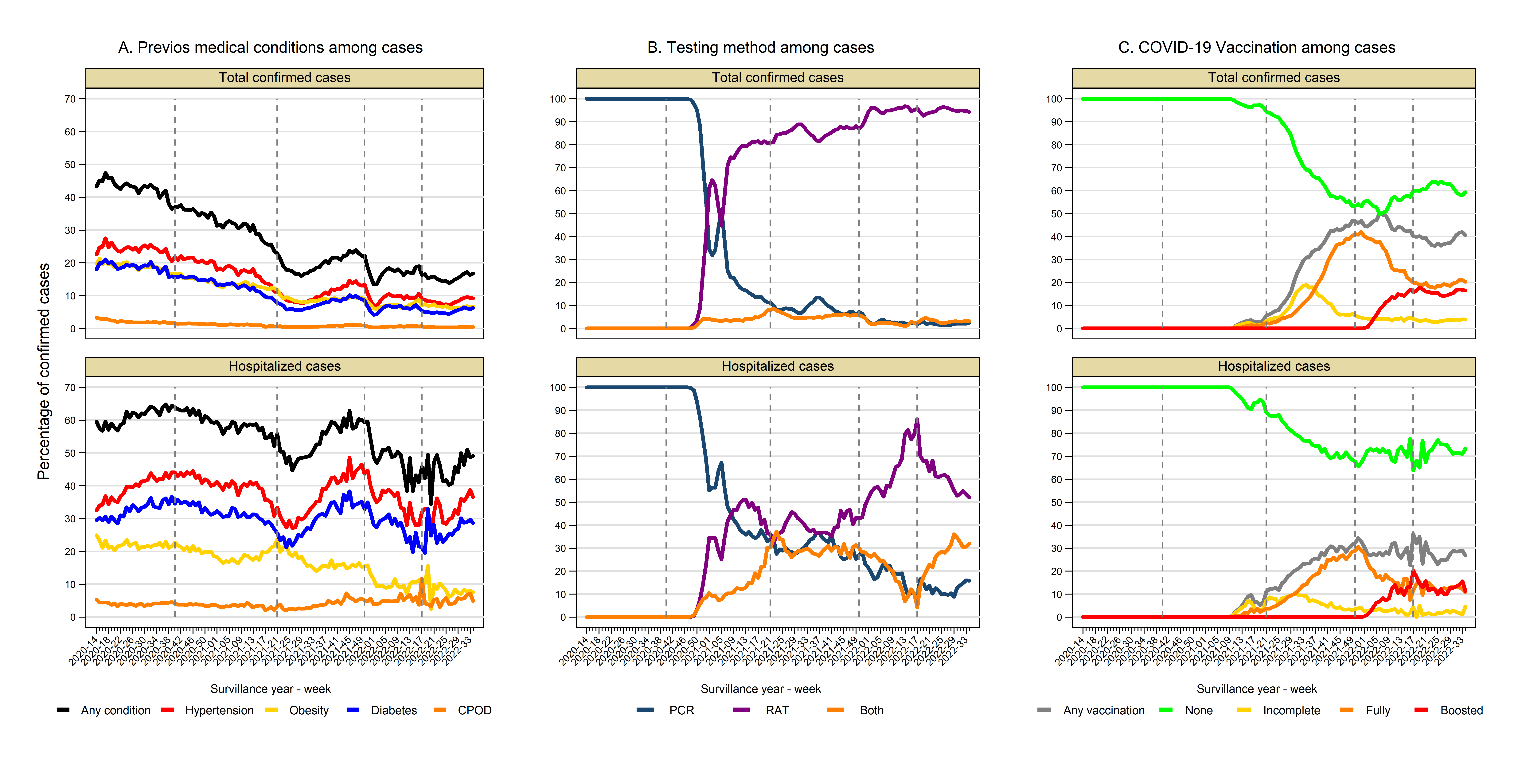


**Supplementary Figure 6 Previous medical conditions, testing methods and COVID-19 vaccination in total confirmed cases and in hospitalized cases.**

*Panel A* shows percentage of confirmed cases with previous medical conditions (black line), including hypertension (red line), obesity (yellow line), diabetes (blue line) and CPOD (orange line).

*Panel B* contains the weekly trend of testing methods among confirmed cases. Blue line corresponds to PCR percentage of confirmed cases, purple line is for rapid antigen test percentage and orange line are for both methods. *Panel C* shows the weekly trend of the percentage of confirmed cases according to the COVID-19 vaccination: gray line for any dose of COVID-19 vaccine, yellow line for incomplete COVID-19 vaccination, orange line for complete COVID-19 vaccination and red line for boosted COVID-19 vaccination. Light green illustrates the percentage of confirmed cases with none COVID-19 vaccine. Upper panels show the distribution among total confirmed cases while lower panels are for hospitalized cases only.

Figures include the onset of symptoms period from week 2020-14 to week 2022-34 (from April 1st, 2020, to August 27th, 2022). Dotted vertical lines represent the separation of the five epidemic waves.

Epidemic waves correspond to the following onset of symptoms periods: the first wave from week 2020-14 to week 2020-40 (from March 29th, 2020 to October 3rd, 2020); the second wave from week 2020-41 until week 2021-21 (from October 4th, 2020 to May 29th, 2021); the third wave from week 2021-22 to week 2021-50 (from May 30th, 2021 until December 18th, 2021); the fourth wave from week 2021-51 to 2022-17 (from December 19th, 2021 to April 30th, 2022); and fifth wave from week 2022-18 to week 2022-34 (from May 1st, 2022 until August 27th, 2022).

.


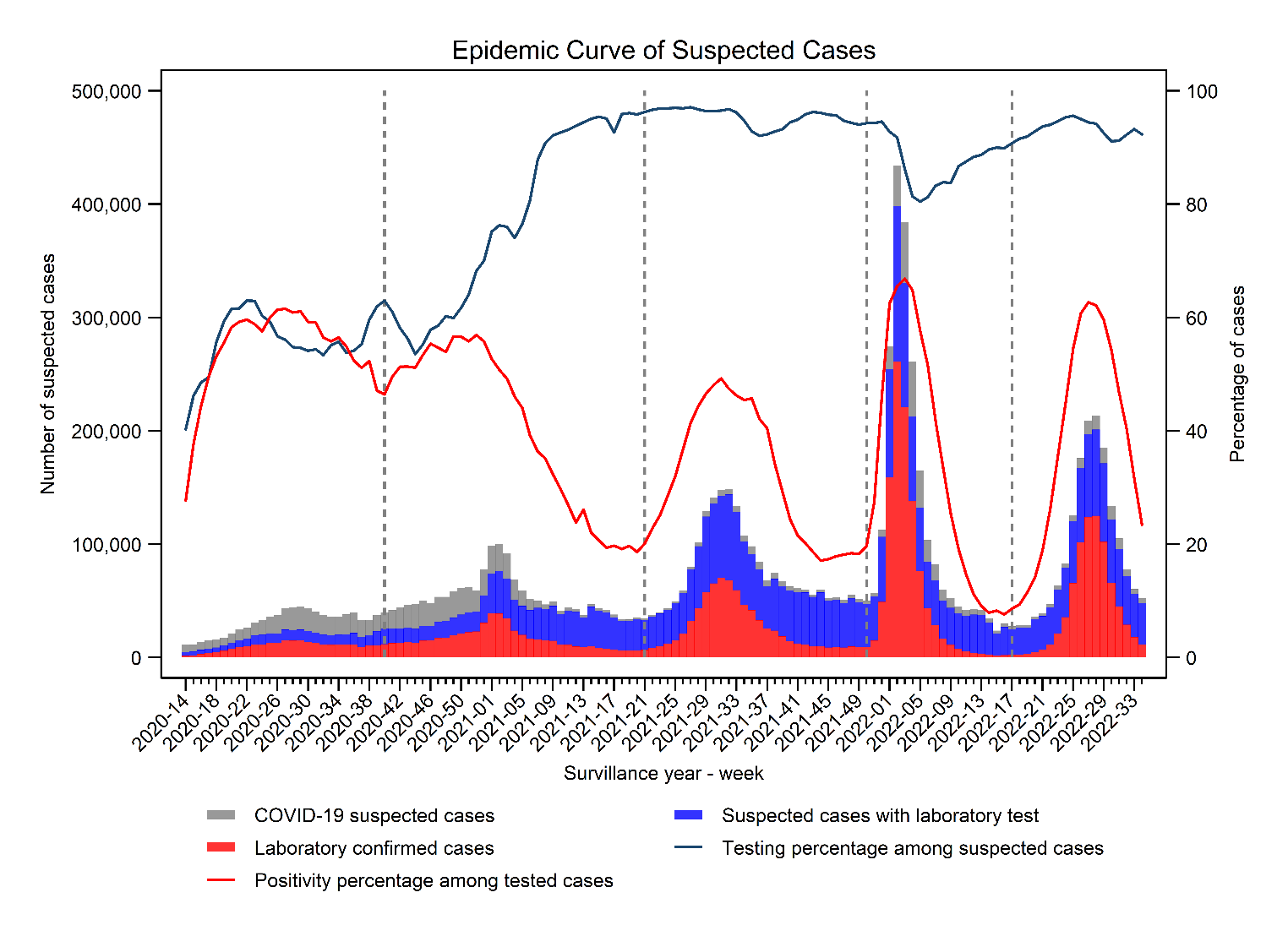


**Supplementary Figure 7. COVID-19 epidemic curve of suspected cases**

This figure shows the weekly number of COVID-19 suspected cases (gray bars). In blue and red bars are displayed the suspected cases with laboratory test and the laboratory confirmed cases, respectively. In blue and red lines are presented the weekly testing percentage among suspected cases and the positivity percentage among tested cases.

Figures include the onset of symptoms period from week 2020-14 to week 2022-34 (from April 1st, 2020, to August 27th, 2022). Dotted vertical lines represent the separation of the five epidemic waves.

Epidemic waves correspond to the following onset of symptoms periods: the first wave from week 2020-14 to week 2020-40 (from March 29th, 2020 to October 3rd, 2020); the second wave from week 2020-41 until week 2021-21 (from October 4th, 2020 to May 29th, 2021); the third wave from week 2021-22 to week 2021-50 (from May 30th, 2021 until December 18th, 2021); the fourth wave from week 2021-51 to 2022-17 (from December 19th, 2021 to April 30th, 2022); and fifth wave from week 2022-18 to week 2022-34 (from May 1st, 2022 until August 27th, 2022).


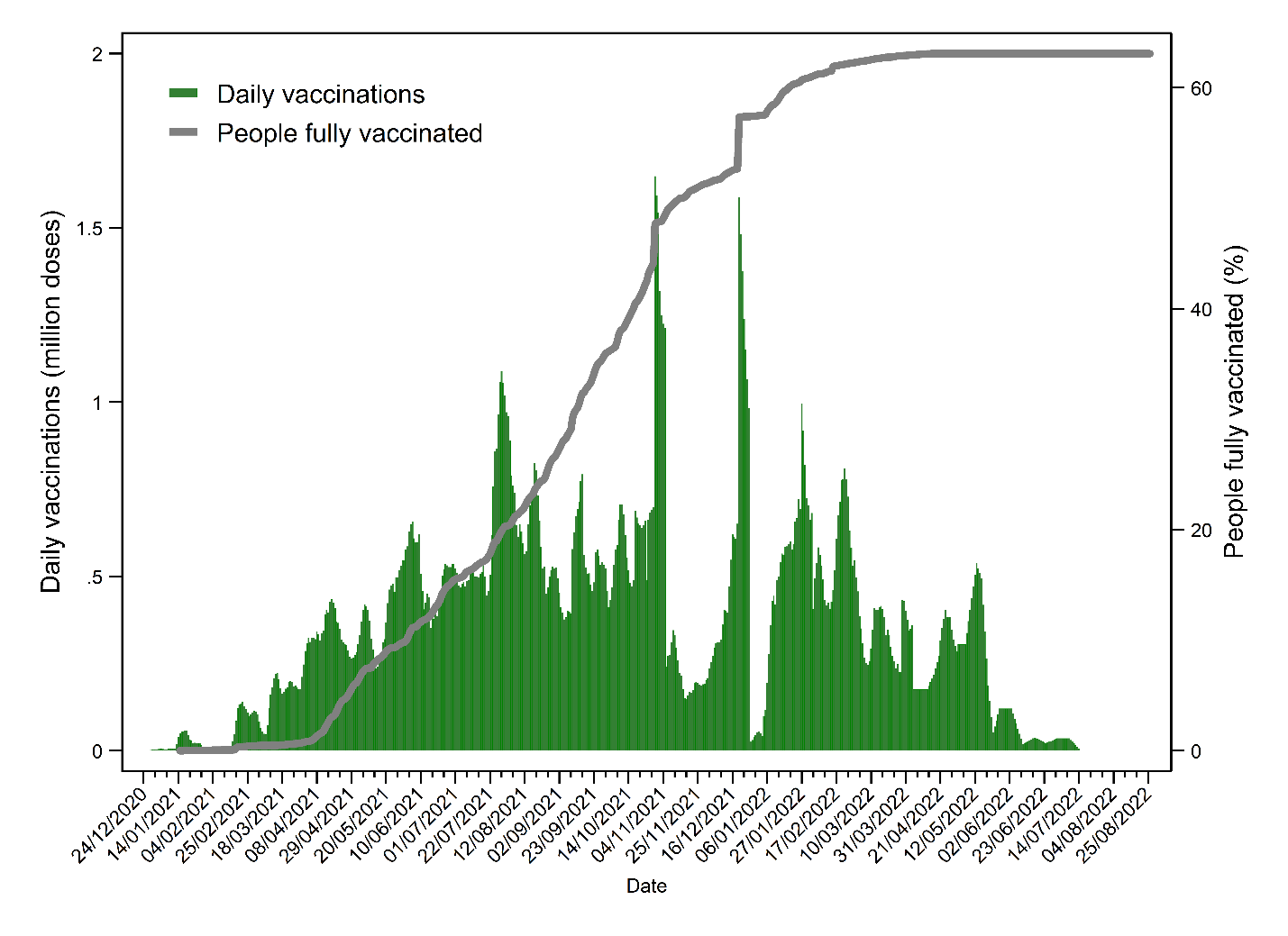


**Supplementary Figure 8. Daily COVID-19 vaccinations and people fully vaccinated in Mexico.**

Green bars represent the daily vaccinations. The gray line in the left axis shows the cumulative percentage of people fully vaccinated. Data were obtained from <https://ourworldindata.org/covid-vaccinations>


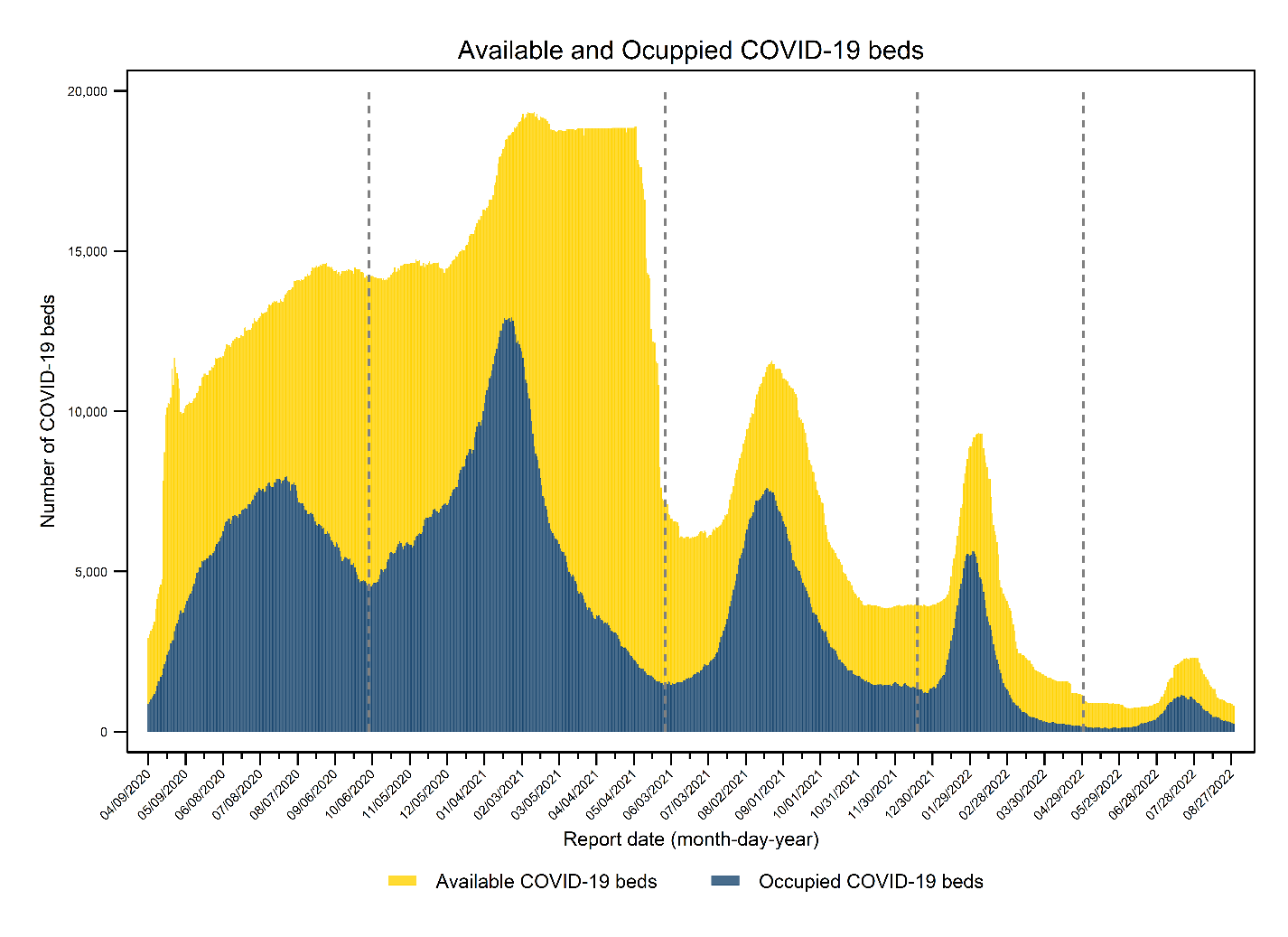


**Supplementary Figure 9. COVID-19 beds available and occupied during the five epidemic waves.**

Figure shows the daily reported number of COVID-19 beds available (yellow bars) and occupied (blue bars) during the five epidemic waves in the IMSS. The period information is from April 9th, 2020, to August 27th, 2022. Dotted vertical lines represent the separation of the five epidemic waves.

In this graph, epidemic waves corresponded to the following report dates: the first wave from April 9th, 2020, to October 3rd, 2020; the second wave from October 4th, 2020, until May 29th, 2021; the third wave from May 30th, 2021, to December 18th, 2021; the fourth wave from December 19th, 2021, to April 30th, 2022; and the fifth wave from May 1st, 2022, to August 27th, 2022. Data was obtained from daily bed occupation report from Virtual Center in Emergencies and Disasters (CVOED) platform.
